# Compound heterozygous variants of ANKFY1 in a child with infantile-onset proteinuria and movement disorder

**DOI:** 10.1093/ckj/sfae124

**Published:** 2024-06-17

**Authors:** Luyan Zhang, Xueqin Cheng, Chunli Wang, Wei Zhou, Bixia Zheng, Aihua Zhang

**Affiliations:** Department of Nephrology, Children's Hospital of Nanjing Medical University, Nanjing, Jiangsu, China; Department of Nephrology, Children's Hospital of Nanjing Medical University, Nanjing, Jiangsu, China; Nanjing Key Laboratory of Pediatrics, Children's Hospital of Nanjing Medical University, Nanjing, Jiangsu, China; Nanjing Key Laboratory of Pediatrics, Children's Hospital of Nanjing Medical University, Nanjing, Jiangsu, China; Nanjing Key Laboratory of Pediatrics, Children's Hospital of Nanjing Medical University, Nanjing, Jiangsu, China; Department of Nephrology, Children's Hospital of Nanjing Medical University, Nanjing, Jiangsu, China

**Keywords:** *ANKFY1*, movement disorder, proteinuria, whole-exome sequencing

## Abstract

The ANKFY1 gene encodes a protein that belongs to double zinc finger proteins involved in endocytosis. Only one family with steroid-resistant nephrotic syndrome has been reported carrying a homozygous variant in ANKFY1 so far. Here we describe the second case where a 13-year-old boy presented with infantile-onset proteinuria and movement disorder. Whole-exome sequencing showed compound heterozygous variants (NM_001330063.2: c.2753C>G; p.Ser918Ter, and c.3287–11_3287–10del) in ANKFY1. *In vitro* functional study revealed the two variants led to reduced protein expression level of ANKFY1. This is the first case of co-existence of renal and nervous system phenotypes in a child with variants in ANKFY1, suggesting that bi-allelic variants in ANKFY1 might be associated with a new neuro-renal syndrome.

## BACKGROUND

ANKFY1 encodes a Rab5-GTP-interacting protein, which localizes to endosomes and is critical in endocytosis. It is highly expressed in the adult human brain with intermediate expression in various other tissues, including the kidney [1, 2]. Ankfy1 knockout mice with a pure genetic background were lethal in embryos, while Ankfy1^+/^^−^ heterozygous mice displayed an abnormal gait with progressive motor and cerebellar nerve dysfunction [3]. Additionally, Ankfy1 has been implicated in the maintenance of cerebellar Purkinje cells [4]. Therefore, variants in the ANKFY1 may be associated with the phenotype in central nervous systems. However, ANKFY1-related disorder in humans is very rare to date. Only one study has reported an ANKFY1 variant in familial SRNS without any nervous system involvement so far [2]. This study presents the second case of one boy who had compound heterozygous variants in the ANKFY1 gene.

## CASE REPORT

A 13-year-old boy was admitted to our hospital because of persistent proteinuria and fever. Proteinuria was initially detected at 5 months, but no specific treatment was administered. At the age of 3, he received corticosteroid therapy at a local hospital, but proteinuria did not improve after 6 months of oral medication. Since then, proteinuria had remained at a level of −∼+, but would aggravate to ++∼+++ after fatigue or infection. The proband experienced developmental delays after birth, lifting his head at 5 months, and was diagnosed with mild cerebral palsy at 1 year of age. He underwent rehabilitation at a local hospital, and starting to walk at 15 months. His parents were healthy and there was no family history of consanguinity, kidney disease, or hereditary disorder.

**Figure 1:**
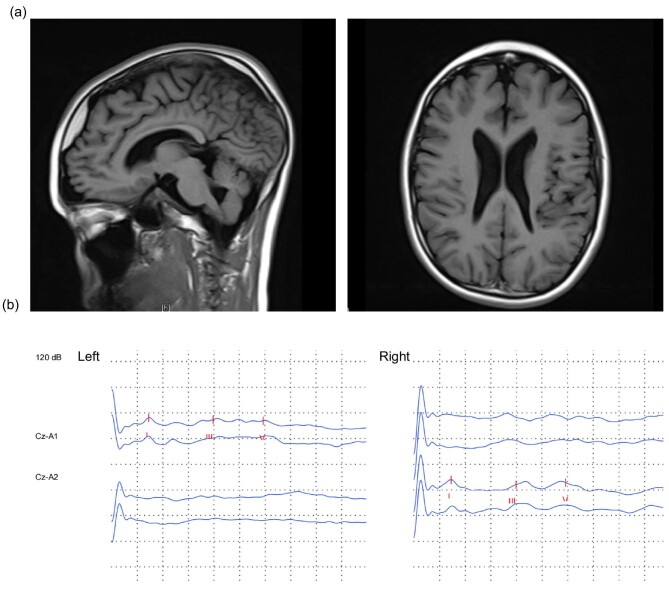
Neurologic features of the proband. (**a**) The brain T1-weight MRI detected thin corpus callosum and widening of the sulci of the brain (left panel), enlarged ventricles with a small possible malacia near the anterior horn of the left lateral ventricle (right panel). (**b**) Recording of brainstem auditory evoked potentials with the respective latencies for waves I, III, and V, of inter-peak interval values I–III, III–V, and l–V, for the proband. The latency of bilateral waves III and V and inter-wave interval values I–III and I–V were slightly prolonged.

The patient's height, measured at 157 cm, and weight, recorded as 41 kg during the physical examination, were within established clinical standards. While his intelligence was normal, he exhibited a thickened tongue, unclear speech, and difficulties in communication. Diminished muscle strength at level 4 was noted, with normal muscle tone. Additionally, he manifested an abnormal gait, notably tiptoeing with his right foot during ambulation. He had poor motor coordination and easily fell while walking, which impedes his engagement in physical activities at school. All other aspects of the physical examination were normal. Serial detection of urinary microprotein indicated an increase in low-molecular-weight protein fraction, with α1 microglobulin of 127 mg/l, microalbumin of 159 mg/l, and IgG 42.3 mg/l, suggesting renal tubular proteinuria. Serum albumin levels and urine calcium creatinine ratio were normal. Urinary ultrasound suggested that both kidneys were of normal size with small crystals. The cranial MRI disclosed a small softening lesion adjacent to the left frontal horn of the lateral ventricle, a relatively thin corpus callosum, fullness in both lateral ventricles, and widened cerebral sulci (Fig. 1a). Electromyography revealed non-active, mildly myogenic alterations. The results of brainstem auditory evoked potentials indicated a mild prolongation in bilateral central auditory pathway conduction time (Fig. 1b). Renal biopsy was declined by parents. After excluding other infectious and metabolic diseases, the patient was managed with benazepril. The patient has been under regular follow-up and has remained in good clinical condition.

**Figure 2:**
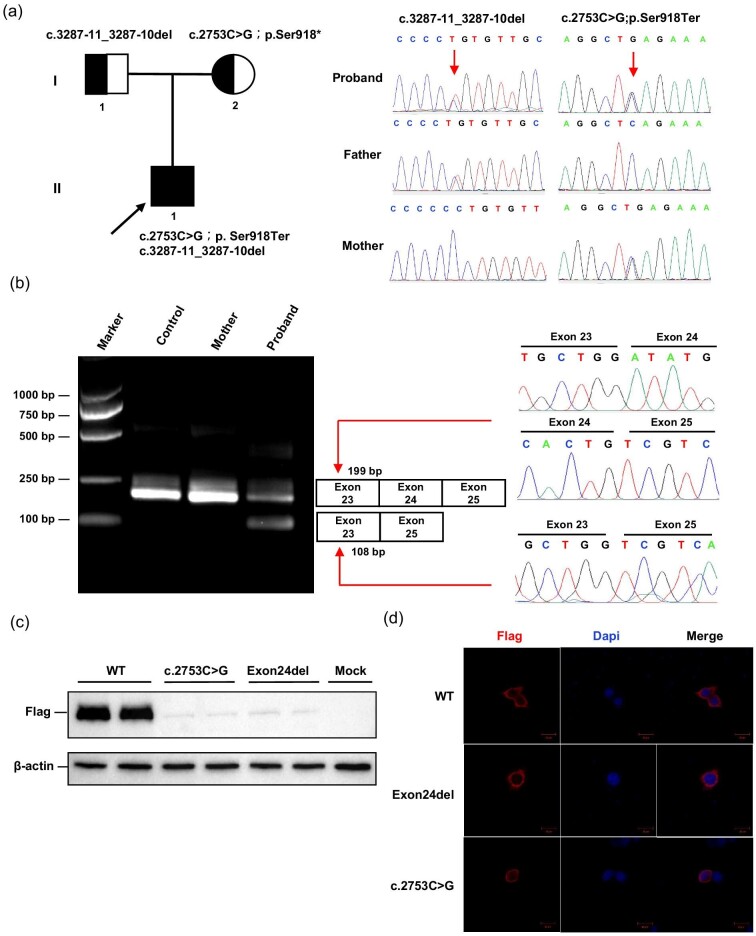
Genetic analysis of the proband. (**a**) Whole-exome sequencing identified two compound heterozygous variants (NM_001 330 063: c2753C>G; p.Ser918Ter, and c.3287–11_3287–10del) in the ANKFY1 gene. Sanger sequencing showed that two variants were maternal and paternal, respectively. (**b**) Gel electrophoresis of reverse transcription PCR fragments *in vivo* showed that c.3287–11_c.3287–10delCT caused abnormal mRNA splicing leading to two transcripts differed by about 108 bp. Sequence analysis of the patient’s mRNA derived transcription PCR products showed that the shorter transcript lacked a sequence corresponding to exon 24 of the ANKFY1 gene. (**c**) 293T cells were transfected with the indicated expression constructs and analyzed by western blot for expression of the two variants. Both variants led to reduced expression of ANKFY1. (**d**) 293T cells were transfected with the indicated expression constructs and immunofluorescence demonstrated that neither variant changed the localization of the protein.

Whole-exome sequencing identified two compound heterozygous variants (NM_001330063.2: c.2753C>G; p.Ser918Ter, and c.3287–11_3287–10del) in *ANKFY1* (Table 1). Sanger sequencing showed the two variants were maternal and paternal, respectively (Fig. 2a). No additional causative variants in known kidney or nervous-related pathogenic gene were identified. The variant c.2753C>G led to the early termination of protein translation encoded by ANKFY1. To verify whether the c.3287–11_3287–10del variant affected mRNA splicing, the patient's and his mother's RNA were extracted from peripheral leukocytes. Using primers mapping on exon 23 and exon 25 for the nested PCR, we obtained two PCR products in the patient and one single product in the control and his mother. Sequencing analysis revealed that the 199 bp amplified product corresponds to the full-length fragment, while the 108 bp band results from an in-frame deletion of exon 24 (Fig. 2b). The effect of the c.2753C>G and deletion of exon 24 variants on protein expression and localization was evaluated by transient overexpression in HEK293 cells (Supplementary methods). Both variants led to reduced protein expression level of ANKFY1 but did not alter its localization (Fig. 2c, d).

**Table 1:** Comprehensive information on new variants in ANKFY1.

Transcript number	Gene	hg19 Pos	c.Change	p.Change	SNP ID	gnomAD (hom/het/allele count)	Mutation taster	CADD
NM_001330063	ANKFY1	chr17:4080443	c.2753C>G	p.Ser918Ter	NA	NA	disease causing	deleterious (41.0)
NM_001330063	ANKFY1	chr17:4072592–4072593	c.3287–11_3287–10del	p.Val1096AspfsTer20	rs1371991279	0/4/184092	NA	NA

## DISCUSSION

ANKFY1 encodes a gene involved in endocytosis, disruption of which has been implicated in the pathophysiology of proteinuria in chronic kidney disease such as SRNS and Dent disease [2, 5]. ANKFY1 gene shows moderate expression in kidney with higher expression in tubules [1]. The proband present with early-onset proteinuria, which can be easily confused with glomerular diseases, such as nephrotic syndrome. However, combined with normal albumin levels, urine examination and urinary ultrasound, renal tubular disease such as Dent disease should be considered first. However, the urine calcium creatinine ratio was normal and no variant in Dent disease associated genes was identified. Since the patient's family declined a renal biopsy, the nature of the patient's renal disease remains undetermined.

Additionally, as mentioned in the background, ANKFY1 is suggested to be involved in nervous system development [3, 4]. Although brain MRI did not reveal significant abnormalities in our patient, the observed movement disorder aligns with the abnormalities in previous studies.

In summary, we identified compound heterozygous variants in the ANKFY1 gene in a patient presenting with both proteinuria and neurological abnormalities. Further evidence and confirmation are needed to elucidate the role of ANKFY1 gene variants in the development of renal and neurological abnormalities.

## ETHICAL APPROVAL

This study protocol was reviewed and approved by the ethics committee of Children's Hospital of Nanjing Medical University, approval number [202306015-1].

## PATIENT CONSENT

Written informed consents were obtained from the participants' parent/legal guardian to participate in the study.

## Supplementary Material

sfae124_Supplemental_File

## Data Availability

All data generated or analyzed during this study are included in this article. Further enquiries can be directed to the corresponding author.
