# Supplementary material for: Compound heterozygous variants of ANKFY1 in a child with infantile-onset proteinuria and movement disorder
Source: Clin Kidney J. 2024 Jun 17;17(6):sfae124. doi: 10.1093/ckj/sfae124 (PMC11194482; doi:10.1093/ckj/sfae124)
Supplement: sfae124_Supplemental_File [file sfae124_supplemental_file.pdf]

Supplementary Method

Plasmid

The full-length cDNA of ANKFY1 inserted into the pcDNA3.1-3xFlag-N vector was obtained from Youbio. Constructs carrying mutant alleles c.2753C>G or deletion of exon 24 were generated from this plasmid. All of the plasmids were confirmed by Sanger sequencing.

Cell culture and transfection

The HEK293 cells were cultured at 37 °C in Dulbecco's modified Eagle's medium (DMEM) supplemented with 10% (vol/vol) fetal bovine serum in a 5% CO_2 incubator. Transfection of HEK293 cells was conducted using Polyjet^{TM} Reagent (Signagen Laboratories, SL100688) according to the manufacturer's instructions, either with the pcDNA3.1 empty vector or plasmids encoding ANKFY1 wild type or two variants. The growth medium was replaced 6 hours post-transfection.

Western blot

RIPA Lysis Buffer (Beyotime, P0013B) containing 1% protease inhibitor was applied, followed by cell dissociation on ice for 30 minutes and centrifugation at 12,000 rpm for 15 minutes at 4 °C. Protein content was quantified using the bicinchoninic acid method (Beyotime, P0012). Equivalent amount of protein (30 μg per lane) was loaded and separated by SDS-PAGE. The protein was transferred to a polyvinylidene fluoride (PVDF) membrane, blocked with 5% skim milk for 1 h at 37°C. The primary antibodies included anti-Flag (1:1000, Sigma-Aldrich, F1804), and anti- Beta Actin (1:20,000, Proteintech, 66009-1-Ig). Following overnight incubation at 4°C in the primary antibody solution, the membranes were washed three times with TBST. Secondary antibody used was HRP-labeled Goat Anti-Mouse IgG (1:200, Beyotime, A0216). After incubation at 37 °C for 1 h, the membranes were washed three times in TBST. Chemiluminescent reagent (TIANGEN, Y2117) was used to visualize the signals.
